# Supplementary material for: Spindle architecture constrains karyotype in budding yeast
Source: bioRxiv. 2023 Oct 25:2023.10.25.563899. Preprint. [Version 1] doi: 10.1101/2023.10.25.563899 (PMC10634821; doi:10.1101/2023.10.25.563899)
Supplement: Supplementary file 1 [file NIHPP2023.10.25.563899v1-supplement-7.pdf]

bioRxiv preprint doi: https://doi.org/10.1101/2023.10.25.563899; this version posted October 25, 2023. The copyright holder for this preprint (which was not certified by peer review) is the author/funder, who has granted bioRxiv a license to display the preprint in perpetuity. It is made available under aCC-BY 4.0 International license.

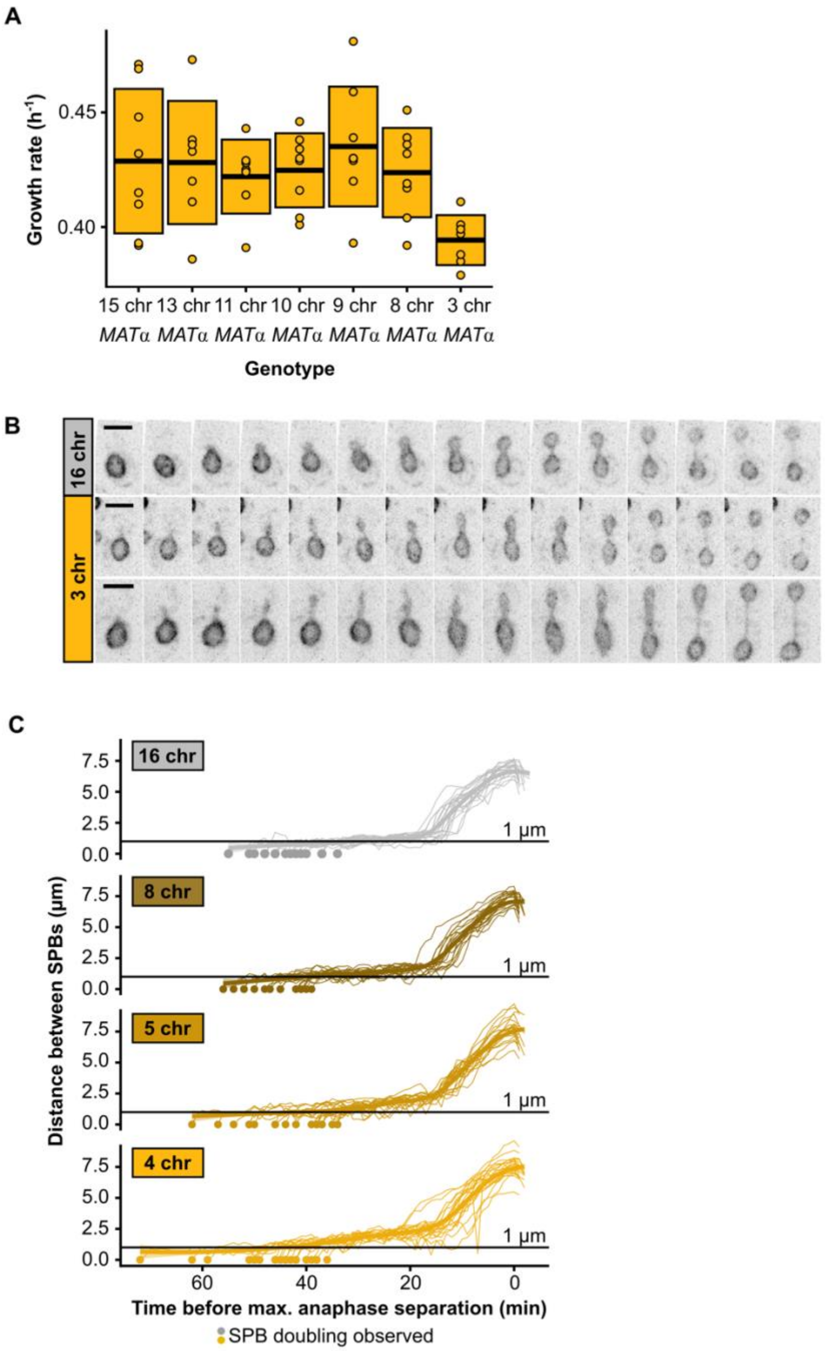

Fig. S1.

Growth and mitotic defects in fused-chromosome strains. (A) Maximum growth rates of fused-chromosome strains on synthetic complete medium with 2% dextrose (SCD). Boxes show the means and standard deviation. **(B)** Montage of nuclear envelope (Hmg1-mCherry) dynamics over time. Scale bar = 5 µm, intervals are 1 min. **(C)** Distance between SPBs over time. For normalization, the time point with maximal SPB separation during anaphase was set to zero.

bioRxiv preprint doi: https://doi.org/10.1101/2023.10.25.563899; this version posted October 25, 2023. The copyright holder for this preprint (which was not certified by peer review) is the author/funder, who has granted bioRxiv a license to display the preprint in perpetuity. It is made available under aCC-BY 4.0 International license.

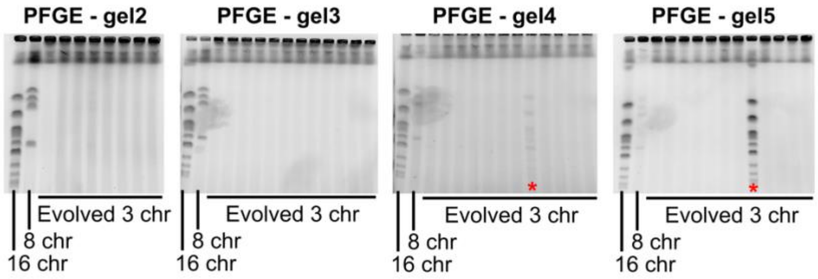

Fig. S2.

PFGE of evolved strains shows no chromosome fission. PFGE gels showing the karyotype of the evolved 3-chromosome populations. Red stars indicate populations with cross-contamination of a wild-type strain. Chromosome numbers of clones isolated from these populations were double-checked before ploidy determination and sequencing.

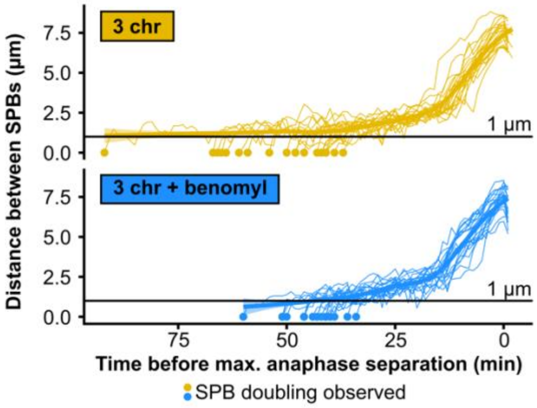

Fig. S3.

Distance between SPBs over time in benomyl-treated cells. For normalization, the time point with maximal SPB separation during anaphase was set to zero.

bioRxiv preprint doi: https://doi.org/10.1101/2023.10.25.563899; this version posted October 25, 2023. The copyright holder for this preprint (which was not certified by peer review) is the author/funder, who has granted bioRxiv a license to display the preprint in perpetuity. It is made available under aCC-BY 4.0 International license.

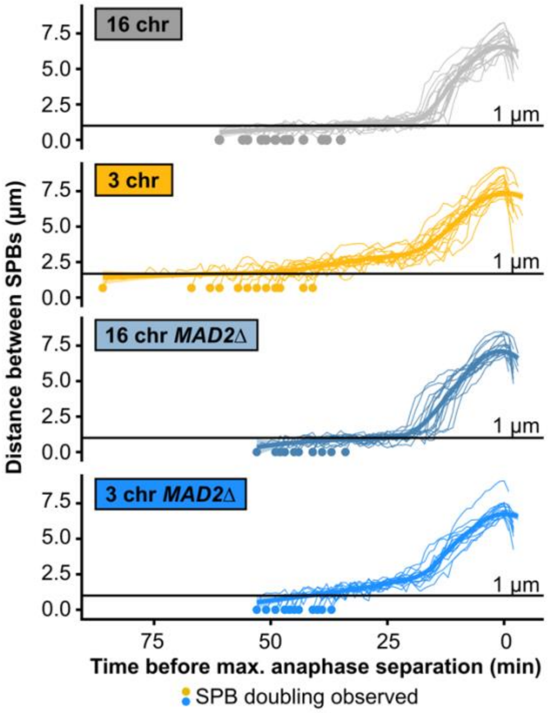

Fig. S4.

Distance between SPBs over time in *MAD2*Δ cells. For normalization, the time point with maximal SPB separation during anaphase was set to zero.
